# Non-antibiotic Approaches for Disease Prevention and Control in Nursery Pigs: A Scoping Review

**DOI:** 10.3389/fvets.2021.620347

**Published:** 2021-04-21

**Authors:** Lee V. Wisener, Jan M. Sargeant, Terri L. O'Sullivan, Annette M. O'Connor, Scott A. McEwen, Mark Reist, Katheryn J. Churchill

**Affiliations:** ^1^Department of Population Medicine, Ontario Veterinary College, University of Guelph, Guelph, ON, Canada; ^2^Centre for Public Health and Zoonoses, University of Guelph, Guelph, ON, Canada; ^3^Department of Large Animal Clinical Sciences, College of Veterinary Medicine, Michigan State University, East Lansing, MI, United States

**Keywords:** antibiotic stewardship, disease control, disease prevention, non-antibioic approaches, nursery pigs, scoping review

## Abstract

Swine producers are encouraged to practice antibiotic stewardship by reducing their use of antibiotics belonging to classes of medical importance to humans. We conducted a scoping review of non-antibiotic approaches in the form of products or management practices that might prevent or control disease and thus reduce the need for antibiotics in nursery pigs. Our objectives were to systematically describe the research on this broad topic for the North American context, identify specific topics that could feasibly support systematic reviews, and identify knowledge gaps. A search of multiple databases identified 11,316 articles and proceedings for relevance screening. From these, 441 eligible clinical trials and observational studies were charted. The majority were clinical trials (94%). Study results from EU countries were mostly communicated through journal articles, whereas study results from the USA were mostly communicated through conference proceedings. Interventions and health outcomes were diverse. The two most frequent intervention categories were feed additives and piglet vaccines. The three most frequent outcomes reported were diarrhea, mortality, and indices of vaccine immunity. There were 13 specific topics comprising various feed additives and vaccines that might feasibly support systematic reviews. There were relatively few studies in which interventions were compared with antibiotic comparison groups and relatively few studies evaluating management practices.

## Introduction

Antimicrobial resistance (AMR) poses a serious threat to advances in modern human medicine, livestock health and production, and animal welfare (1, 2). There are limited or few alternative treatment options in patients infected with pathogens resistant to medically important antibiotics, particularly those with resistance to critically important antibiotics (3). The World Health Organization (WHO), the World Organization for Animal Health (OIE), and the Food and Agriculture Organization of the United Nations (FAO) regard antimicrobial use (AMU) in as a significant driver of AMR in humans and animals alike (2, 4, 5). In a tripartite “One Health” approach, these major global institutions have called for a worldwide effort to reduce inappropriate and unnecessary AMU in all sectors (2, 4, 5). The WHO and the OIE have categorized antibiotic classes according to their importance for human and animal health, respectively (3, 6). Categorization of antibiotic classes is useful for prioritization of strategies to limit AMR, such as antibiotic stewardship. To help achieve this goal, the WHO has published guidelines recommending certain restrictions on the use of medically important antibiotics in non-human sectors (7). Food production sectors use antibiotics of importance to humans that are the same or belong to the same antibiotic class as those used in human medicine for animal disease treatment or prevention in vulnerable individuals or groups (3, 5, 8).

Worldwide, nations are heeding the call for reduced antibiotic use in food production through regulation and industry guidelines. For example, in the USA, the US Food and Drug Administration Center for Veterinary Medicine promotes prudent use of livestock antibiotics belonging to classes of importance to humans by requiring they be administered with veterinary oversight and be limited to the purpose of assuring animal health. The use of medically important antibiotics in healthy animals for growth promotion was prohibited by 2017 in the USA (9). In Canada, similar regulations enacted by December 2018 eliminated the over-the-counter use of antibiotics belonging to classes of medical importance to humans by requiring veterinary oversight for administration of these antibiotics by injection or by addition to feed or water (10). In the European Union (EU), the use of antibiotics in feeds for growth promotion has been banned since 2006 (11). In their systematic review of antibiotic use in swine production from 2000 to 2017, Lekagul et al. (12) reported that there was geographical variation in antibiotic use by types of diseases. Choice of antibiotic was dependent upon the common pathogens associated with age-specific diseases and upon route of administration, typically oral in-feed medication in nursery pigs. Lekagul et al. (12) concluded that medically important antibiotics are still commonly used worldwide for disease prevention and control in swine production, particularly in modern commercial swine production during the suckling piglet and nursery pig stages.

Scoping review methodology is used to systematically map the literature with regard to the extent, range, and nature of existing research of a particular topic area (13, 14). Scoping reviews are also useful as preliminary “reconnaissance” to assess the feasibility of undertaking a full systematic review of a specific topic and to identify gaps in the existing research (13, 14). While scoping reviews are descriptive and broad in nature, systematic reviews aim to address a specific research question by using explicit systematic methods to collate all the evidence that fits pre-specified eligibility criteria while minimizing bias (13, 15). To help inform antibiotic stewardship goals, a scoping review of non-antibiotic approaches to nursery pig health could help researchers advance the knowledge of alternative approaches by indicating topics that could be subject to formal systematic reviews or merit further research. In addition, a scoping review could illuminate current gaps in research on non-antibiotic approaches to nursery pig health for swine industry professionals, swine researchers, and research funding agencies.

The objectives of this scoping review were three-fold: (i) to examine and describe the volume, range, and nature of research on non-antibiotic approaches for disease prevention and control in commercial nursery pig production; (ii) to identify specific topics where available research literature may support systematic reviews; (iii) to identify knowledge gaps in the primary literature on the effectiveness of various non-antibiotic approaches. Summarizing the literature regarding intervention effectiveness was not an objective for this scoping review.

## Materials and Methods

This scoping review followed the framework for scoping reviews as outlined by Arksey and O'Malley (13) using the PRISMA-ScR (i.e., Preferred Reporting Items for Systematic Reviews and Meta-Analyses Extension for Scoping Reviews) guidelines for reporting scoping reviews (16). The registered protocol can be located through UoG Atrium https://atrium.lib.uoguelph.ca/xmlui/handle/10214/12929.

Our review question was as follows: What are the volume and nature of the primary research literature published between 2000 and 2018 that evaluated non-antibiotic interventions (i.e., products such as vaccines or feed additives and management practices such as weaning methods or biosecurity) to prevent or control bacterial and viral illnesses in nursery pig production in North America and regions or countries with similar production conditions? Viral illnesses were included because we presumed that preventing viral infections may reduce secondary bacterial illnesses.

### Expert Stakeholder Engagement

As the volume of literature on the broad topic of non-antibiotic approaches for nursery pig health was potentially very extensive, we sought expert opinion to refine our research question and help to inform our search strategy and study eligibility criteria. More specifically, the stakeholder engagement served six objectives: (i) to identify the countries or regions with similar commercial swine production practices to those of North America, (ii) to select the swine production stage(s) for which non-antibiotic approaches could most effectively reduce total AMU in swine production, (iii) to solicit opinions on the importance of various antimicrobial types to antimicrobial stewardship in the swine industry (e.g., antibiotics belonging to medically important classes used to prevent or control swine diseases, anticoccidials, and anthelmintics), (iv) to identify the health and production outcomes of greatest importance to swine producers, (v) to identify the most important non-antibiotic interventions in the form of specific products (e.g., vaccines, feed additives, medications, or supplements) or specific management practices (e.g., weaning practices, biosecurity, housing, feed type, and restricted feeding); and (vi) to identity any additional outcomes or interventions that we had not included on our initial lists. We also asked the stakeholders to recommend other experts who should be consulted.

The expert opinion was gathered through an anonymous online survey (Qualtrics XM). The respondents selected and ranked options from a list for a particular question and/or responded in an open-ended format. Expert stakeholders who were consulted included representatives within provincial or national Canadian swine industry associations, representatives in provincial agriculture departments, and Canadian scientists engaged in swine agriculture and/or swine veterinary research. We assumed that the responses from stakeholders of Canadian institutions would be representative of experts from the USA and other countries with commercial swine production. Stakeholders' most common responses and suggested additional responses were used to inform the parameters of the search, eligibility criteria, and data items. Ethics approval for this survey was not required, as the results were used strictly to inform the review process and not reported as a finding of the review.

### Eligibility Criteria

Eligible information sources were from North America, EU countries, Australia, and New Zealand, available since 2000, as these sources of evidence were most likely to reflect current commercial swine production systems most similar to those of North America. Eligible publication types included English-language journal articles, conference proceedings, theses, and technical reports. In addition to electronic databases, proceedings were sourced from the American Association of Swine Veterinarians (AASV) Annual Meeting and Pre-conference seminar from 2000 to 2018 and the International Pig Veterinary Society Congress biannual meetings from 2000 to 2016. We included research reports that reported a challenge trial, a clinical trial (i.e., a controlled trial), or observational study of a modifiable intervention, and we reported a health or production outcome in nursery pigs. For purposes of this review, “interventions” also included modifiable risk factors. Consistent with our stakeholder engagement results, interventions related to breed or genetic improvement were not included. Our stakeholder engagement also informed our *a priori* selected outcomes of interest. These included health outcomes of bacterial or viral infections significant to swine health in North America, treatment costs, and measures of performance. Studies evaluating toxicities or parasitic infections were excluded. Non-English information sources were excluded. Quasi-experimental intervention studies designed as “before period” vs. “after period” of the intervention application were excluded.

### Information Sources

To aid in the development and validation of the search, we checked that 25 known relevant citations are included. The primary reviewer (LW) performed the database search from March 27, 2018, to April 19, 2018, using multiple databases hosted by the data platforms of ProQuest, Web of Science, and PubMed (Table 1). Though the database CAB Direct was originally targeted for inclusion, due to technical difficulties, it was not used. The search for proceedings was conducted manually by the primary reviewer and a second reviewer working independently subsequent to obtaining access to the online AASV Swine Information Library through a membership, September 20, 2018.

**Table 1:** Data platform and database information sources used in the scoping review search on non-antibiotic approaches to reduce the need for antibiotics in nursery pig production.

**Data platform**	**Databases**
ProQuest	Agricultural and environmental science AGRICOLA and TOXLINE
	Biological science database (MEDLINE and TOXLINE), dissertations and theses Guelph, ProQuest dissertations and theses
ProQuest	AGRICOLA
PubMed	PubMed (not MEDLINE)
Web of science	Science citation index, conference proceedings citation index–science
Web of science	MEDLINE
AASV	Annual meeting proceedings
AASV	International pig veterinary congress (biannual meetings) proceedings

### Search

The database search was filtered by language (English), date of publication (published between 2000 and date of search in 2018), and by location filters for eligible countries if available. Additional filters were applied as were allowable within the data platform. These included source and document type (i.e., article, proceedings paper, meeting abstract, and thesis), subject or research areas, and Medical Subject Headings (MeSH) or qualifiers. The search terms were limited to the title or abstract in the PubMed and Web of Science platforms or “anywhere except full text” for the ProQuest platform databases. Management of the identified citations was as follows: first, they were imported into the reference manager software EndNote (Clarivate Analytics, Philadelphia, United States); second, they underwent exact match deduplication in EndNote; and third, they were imported into the systematic review software DistillerSR (Evidence Partners, Ottawa, Canada) where they underwent further deduplication based on close matches.

The database search strategy included a string of the population term groupings (e.g., weanling, nursery pig, and starter pig), one of two intervention term groupings, and an outcome term grouping (e.g., health, diarrhea, or growth) with each grouping connected by the Boolean operator “AND” (Table 2). There were two intervention term groupings, one for interventions of interest in the form of a product (e.g., vaccine, feed supplement, or plant extract) and another for interventions in the form of a management practice (e.g., antibiotic-free and late weaning as defined by the study authors, or disinfection). Within each term grouping, terms were combined by the use of the Boolean operator “OR.” The search was conducted by the primary reviewer (LW) in consultation with a University of Guelph research librarian.

**Table 2:** Search terms for non-antibiotic approaches to reduce the need for antibiotics in nursery pig production.

**Groupings**	**Search terms**
Population terms	(Piglet* OR weaner* OR “weaning pig*” OR “weanling pig*” OR “weaner pig*” OR “weaned pig*” OR “weaner stage” OR “weaner phase” OR “nursery pig*” OR “young pig*” OR “younger pig*” OR “early-weaned pig*” OR “late-weaned pig*” OR “nursery-age*” OR “naïve pig*” OR “starter pig*” OR “neonate pig*” OR “neonatal pig*” OR “suckling pig*”)
Product intervention terms	(Antibiotic* OR antimicrobial* OR vaccin* OR immunization OR “sow vacc*” OR “dam vacc*” OR “gilt vacc*” OR “sow immunization” OR “dam immunization” OR “gilt immunization” OR “trace mineral*” OR “essential mineral*” OR “mineral source*” OR “mineral form*” OR Zinc*OR vitamin* OR “dietary acid*” OR “organic acid*” OR “dietary fatty acid*” OR “medium chain fatty acid*” OR acidif* OR “feed enzyme*” OR fermentable OR fermented OR “plant extract*” OR herbal OR seaweed OR spice OR phytogenic OR “dietary lysine” OR “dietary tryptophan” OR lactoferrin OR lysozyme OR L-glutamine OR nutraceutical* OR neutraceutical* OR supplemental OR “dietary supplement*” OR “diet supplement*” OR “feed supplement*” OR “dietary additive*” OR “diet additive*” OR inulin OR oligosaccharide* OR polysaccharide* OR mannan* OR B-glucan* OR probiotic* OR prebiotic* OR synbiotic* OR “direct-fed microbial*” OR “competitive exclusion” OR yeast OR “Saccharomyces cerevisiae” OR “essential oil*” OR “fish meal” OR “blood meal” OR “spray-dried” OR immunoprophylaxis OR immunotherapeutic* OR “egg-yolk antibod*” OR “IgY antibod*” OR bacteriophages OR “antimicrobial peptide*” OR “bovine colostrum” OR “epidermal growth factor*” OR “rare earth” OR clay OR “natural alternative*” OR homeopath*)
Management intervention terms	(“natural pig*” OR “organic swine” OR “organic pig*” OR “natural conditions” OR “non-conventional” OR “antibiotic-free” OR “weaning practice*” OR “weaning method*” OR “weaning procedure*” OR “weaning regime*” OR “weaning system” OR “conventional weaning” OR “weaning age” OR “early weaning” OR “late wean*” OR “age at weaning” OR “creep feed*” OR “stocking” OR crowding OR overcrowding OR “floor space” OR “feeder space” OR “housing system*” OR “housing design*” OR “housing environment*” OR “housing type” OR ventilation OR “air quality” OR co-mingling OR “mingl*” OR “mixed litter” OR mixing OR “batch system” OR “batch management” OR biosecurity OR “sanit*” OR “disinfect*” OR “cleaning” OR hygiene OR “all-in-all-out” OR “pig flow” OR “disease eradication” OR “disease control*” OR “multi-site” OR “liquid feed” OR “liquid diet*” OR “pellet*” OR “low protein” OR “decreased protein” OR “restricted protein” OR “protein restrict*” OR “protein nutrition” OR “protein level” OR “protein source” OR “dietary protein” OR “restricted feed*” OR “feed restrict*” OR “control fed” OR “quality assurance” OR education)
Outcome terms	(health OR immun* OR diarrhea OR diarrhea OR scours OR “colibacillosis” OR “fecal score” OR “clinical response*” OR “clinical parameters” OR “fecal shedding” OR “fecal shedding” OR morbidity OR mortality OR performance OR growth OR “daily weight gain” OR “average daily gain” OR “G:F” OR “gain-to-feed” OR “feed conversion” OR “feed intake” OR “ADG” OR ADFI OR “lightweight gain” OR productivity)

*A search string included the population terms and either the product intervention terms, or the management intervention terms plus the outcome terms connected by the Boolean operator “AND”*.

The online proceedings identified as being potentially relevant based on subject headings and titles were entered onto a Microsoft Excel (2010) spreadsheet for tracking of further screening decisions. Relevant full-text proceedings were entered into the DistillerSR database. Proceedings that were duplicates of journal articles were removed.

### Selection of Sources of Evidence

Our review team consisted of veterinary epidemiologists, one of whom acted as the primary reviewer, a topic expert in swine research (TOS), and two trained MSc epidemiologists who acted as second reviewers. Pre-testing of the relevance screening form was conducted on 100 citations based on title and abstracts. Pre-testing of the data charting was conducted on 25 full-text articles. Using forms created in DistillerSR, two independent reviewers screened and charted the data. Any disagreements were resolved by consensus or a third reviewer. After pre-testing, the level 1 relevance screening form did not change.

However, due to the large volume of literature identified after the first level of relevance screening, three additional relevance screening levels were applied to the titles and abstracts to refine the selection of relevant citations to the literature that was most pertinent to our research question (Appendix 1, level 1–4 relevance screening forms). Level 2 screened by eligible countries or regions by first author address. Level 3 screened by study type and information regarding the challenge pathogen or antigen that was collected, but then challenge trials were excluded from further screening and data charting. Level 3 also screened by pig type (i.e., included only conventional or specific pathogen-free pigs). Level 4 screened by intent of the intervention (i.e., included non-antibiotic interventions for viral or bacterial infections, excluded interventions for mycotoxins and soy allergens) and by eligible diseases (i.e., excluded reportable or rare diseases such as classic swine fever, Aujeszky's disease and foot and mouth disease, and outcomes only of public health impact such as swine hepatitis E virus and methicillin-resistant *Staphylococcus aureus*). At level 4, the articles that only reported a performance outcome without any health outcomes of interest were excluded from data charting. Health outcomes of interest were defined *a priori* and included clinical outcomes [i.e., mortality all-cause, diarrhea, respiratory disease and non-specific morbidity defined as non-diarrheal, non-respiratory non-specific morbidity (e.g., pyrexia, removals, or unthriftiness), or other morbidities such as lameness], surrogate health outcomes such as shedding of clinically important pathogens [i.e., S*almonella* spp., *Campylobacter* spp., enterotoxigenic *Escherichia coli* (ETEC *E. coli*)], and measures of specific and non-specific immunity to vaccines or bacterins (Table 3). In summary, articles that were selected for data charting reported research on non-antibiotic approaches to improve health outcomes of important viral and/or bacterial infections in conventional or specific pathogen-free nursery pigs in North America, EU countries, the UK, New Zealand, or Australia. The additional relevance screening levels 2–4 were a protocol deviation intended to focus the data charting on studies that addressed our research question.

**Table 3:** Description of data charting items for relevant journal articles, technical reports, proceedings, or theses.

**Variable**	**Description of items**
**General study characteristics**	
Study design	Clinical trial (i.e., experimental or field-based trial under conditions of natural exposure), challenge trial (i.e., deliberate exposure to a pathogen or antigen under the control of the investigator), observational study
Study location	Country and region where the study was conducted as stated in the article or if not stated, first author address
Year of publication	Year of publication or year of proceeding
Farm setting	Population farm setting (i.e., experimental research farm, commercial farm, or unclear)
**Detailed trial or observational study characteristics**	
Specific pig population in which the intervention was given	Specific population based on production stage included dams, suckling piglets, nursery pigs
Purpose^a^ of the intervention as stated in the title or objective statement	Disease prevention (i.e., no pre-existing health problems or known exposures), disease treatment (i.e., individual pigs or groups in whole or part or the farm were known to have clinical disease or exposure to viral or bacterial pathogens. In addition, some studies included performance (e.g., feed intake, growth or body weight, feed efficiency)
Non-antibiotic interventions in the form of a product or management practice or risk factor studied	*Products*: Piglet vaccines, maternal vaccination, non-antibiotic feed or water additive including the addition of specific dietary components, non-antibiotic medication (e.g., any medication, vitamin, mineral, antibodies, etc. administered directly to an individual). Combination products used as interventions that contained both an antibiotic [e.g., Zinc Oxide (ZnO) plus an antibiotic] were excluded. *Management:* Feeding regime as amount or schedule (e.g., protein level, creep feeding, restricted feeding); diet type or format (e.g., pelleted vs. mash, fermented feeds, complexity of feeds); weaning method or weaning stage as defined by the authors (e.g., early vs. late); biosecurity (e.g., comingling, mixing, introductions, animal movements); housing, flooring or feeders (e.g., animal density, feed troughs and water supply factors, flooring); air quality; producer education
Comparison groups	No treatment or conventional practice comparison, placebo or sham, different level or form of treatment, antibiotic and/or ZnO^b^
Health outcomes of interest reported	Mortality (i.e., piglet deaths in absolute terms, deaths per time period, excess deaths, or other metric); clinical diarrhea (e.g., scours, fecal consistency, or fecal score); clinical respiratory disease; non-diarrheal, non-respiratory non-specific morbidity (e.g., fever, removals or unthriftiness) or other morbidities such as lameness; treatment for illnesses or antibiotic use; pathology or lesions; fecal shedding of specific swine pathogens; measures of specific and non-specific immunity and infection (i.e., serology, cell mediated immunity, viremia, PCR, immune markers such as acute-phase proteins, or tumor necrosis factor (TNF)
Other outcomes measured	None, performance outcomes (i.e., feed intake, growth or body weight, or feed efficiency), farm economics or treatment costs, diet digestibility, gastrointestinal microflora, gastrointestinal morphology
Study size	Number of study subjects in each study at the hierarchical level of the analysis (e.g., individual, pen or group, herd or farm)

a *Studies in which the purpose included both prevention and treatment were counted as disease control in results*.

b *Some studies compared a non-antibiotic intervention group to a zinc oxide comparison group while other studies compared a zinc oxide treatment group to a no-treatment control group, antibiotic or other treatment comparison group*.

### Data Charting Process

Data charting of full-text articles (i.e., journal articles, technical reports, theses, and conference proceedings) was conducted by both the primary reviewer and a second reviewer working independently using a form in DistillerSR (Appendix 2, Data charting form). Any disagreements were resolved by consensus or a third reviewer. Data were charted at the individual study level.

We focused the data charting on clinical trials and observational studies that reported a health outcome of interest in the nursery stage of production. However, for studies that reported a health outcome of interest, additional data charting related to other health outcomes and non-health outcomes was completed. A further protocol deviation included an additional question regarding the stage of production at which the intervention was applied (e.g., reproduction, suckling, or nursery).

### Data Items

We charted data for the publication type and for the following study characteristics: study design, study location and year of publication or conference year, study size, and farm settings of the study population (i.e., experimental farm vs. commercial farm). In addition, we charted data for the following: production stage(s) of animals receiving the intervention, purpose of intervention as disease prevention, and/or treatment, and/or performance; specific intervention evaluated; comparison group(s); health outcomes of interest reported; other outcomes reported; and study size and the hierarchical level at which the outcome was measured (i.e., individual, group/pen, or herd/farm) (Table 3). Data were charted using preselected response options with an added text box for additional responses or clarification for the interventions, comparator groups, and outcomes reported (Appendix 2).

In this review, interventions such as non-antibiotic medication, vitamin, mineral, or antibody given directly to individual animals *via* injection or oral bolus were charted as a “medication,” whereas the same intervention given to groups of animals *via* feed was charted as a “feed additive.” There were two data charting options related to measures of immunity as an outcome, one specifically for vaccine immunity and another for non-vaccine immunity.

Data charted regarding the purpose(s) of the intervention were based on information in the title or objective statement. Prevention was selected if the herd or group of pigs showed no clinical or subclinical evidence of disease or infection, whereas treatment was selected if the pigs as individuals or groups in part or whole showed evidence of infection, or were known to be exposed. Although we charted data for disease prevention and/or treatment, in the results, we reported disease control for those studies that described an intervention given for both purposes, treatment and prevention (i.e., the intervention was given to groups of pigs assumed to comprise both healthy and clinically or subclinically affected or exposed pigs). Definitions for disease prevention, treatment, control, and pig performance were based on those provided by the United States Government Accountability Office (GAO) (17) and the American Veterinary Medical Association (18).

Comparison groups that were not clearly stated as “no treatment” or “conventional practice” were charted as comparison groups that received a “different form or level” of the intervention or exposure. Studies could be charted with multiple types of comparison groups (i.e., both a “no treatment” control group and “different form or level” if additional comparison groups received various levels or forms of the treatment or exposure). Zinc oxide (ZnO) was charted as an intervention when it was the study intervention of interest and charted as a comparison group when another non-antibiotic intervention of interest was compared with zinc treatment.

To meet our second objective of identifying specific intervention topics that could be combined for systematic reviews, we considered only clinical trials with clinically important outcomes. These included mortality; non-diarrheal, non-respiratory non-specific morbidity, or other morbidities such as lameness; diarrhea or fecal score; respiratory disease; and treatment for illness or antibiotic use. Thus, not all health outcomes of interest were considered as clinically important outcomes. The criteria for selection of topic areas as potentially extensive enough for systematic reviews were arbitrarily set at a minimum of 10 clinical trials reporting a similar intervention in the same population (e.g., amino acids in nursery pig feed, specific piglet vaccines, and/or dam vaccines) and one or more clinically important outcomes, though not necessarily the same clinically important outcome among all clinical trials for the specific topic area.

### Synthesis of Results

Data charted in DistillerSR were entered into a database in Stata 15.1 (College Station, Texas, USA). These data were summarized descriptively and presented in the form of tables and figures in accordance with our stated data charting scheme. So as to emphasize the research with the highest evidentiary value, we present detailed results for articles that reported clinical trials and observational studies (19). For the challenge trials, we presented only details of the types of challenges evaluated as obtained during level 3 screening.

## Results

### Expert Stakeholder Engagement

A total of 73 experts were invited to respond to the survey, of which 33 responded (45%). We incorporated the stakeholder input into our search strategy and data charting items. There were no suggested publications that were not identified through our search.

### Selection of Sources of Evidence

**Figure 1:**
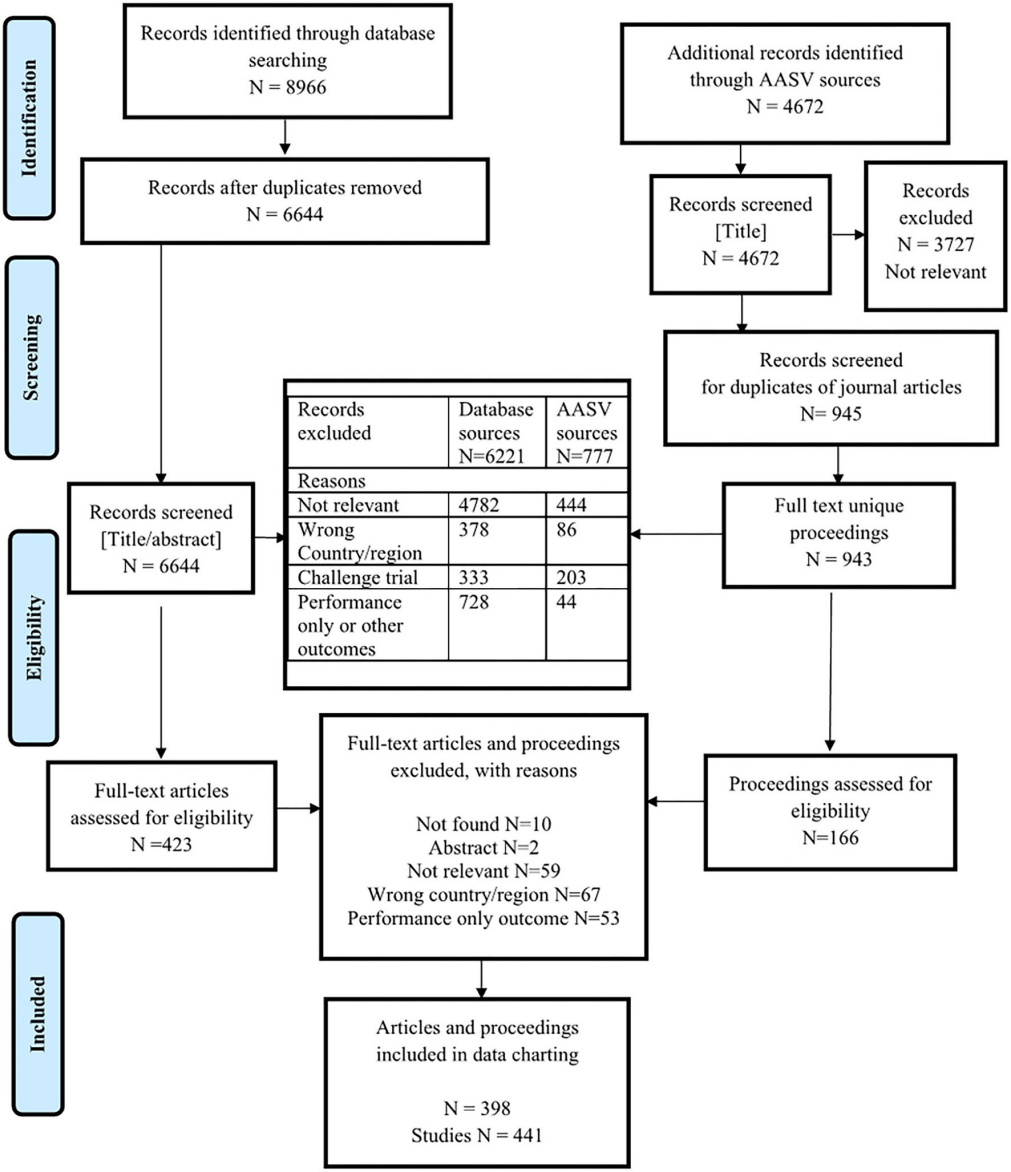
Preferred Reporting Items for Systematic Reviews and Meta-Analyses (PRISMA) flow diagram of citations from literature search through to relevance screening and data extraction.

There were 11,316 unique citations screened for eligibility: 6,644 were from the database search and 4,672 were from the gray literature proceedings of the AASV Swine Information Library sources (Figure 1). Two proceedings that were duplicates of journal articles were removed (Figure 1). A total of 536 challenge trials were identified at level 3 screening of database sources and at full-text screening of proceedings. A description of the types of challenge agents is presented in Appendix 3. A total of 772 journal articles and proceedings that described clinical trials or observational studies but only reported a performance outcome or other outcome without reporting any health outcome of interest were excluded at level 4 screening of the database sources and full-text screening of proceedings. In total, 589 citations (5%) describing clinical trials or observational studies and reporting a health outcome of interest were screened for eligibility based on full text. Among these, 398 were eligible for data charting. Thirty-four eligible articles (8.5%) described one or more studies; in total, there were 441 relevant clinical trial or observational studies included for data charting.

### Characteristics of Sources of Evidence

The majority of eligible studies were clinical trials (*n* = 414, 94%). The remainder were observational studies (*n* = 27, 6%). The observational studies were conducted exclusively using animals living in commercial farm settings, whereas the clinical trials were conducted using animals living in experimental farm settings (*n* = 206, 50%) and in commercial farm settings (*n* = 182, 44%). The farm settings were unclear for 26 (6%) clinical trials, of which 20 were reported in proceedings.

The majority of the studies were conducted in EU countries or the UK (*n* = 284, 64%) followed by the USA (*n* = 110, 25%). There were 17 studies conducted in Australia or New Zealand. The five EU countries with the greatest numbers of studies were Spain, Denmark, Poland, Germany, and France. The five specific states of the USA with the greatest numbers of studies were Minnesota, Iowa, North Carolina, Illinois, and Nebraska. Among the 27 observational studies, 19 were conducted in EU countries and five were conducted in Canada. Among the 414 clinical trials, 265 were conducted in EU countries or the UK, 108 were conducted in the USA, and 25 were conducted in Canada.

The body of eligible studies (*n* = 441) was composed of published articles (*n* = 297, 67%) and proceedings (*n* = 144, 33%). The 284 studies reported from EU countries and the UK were mostly communicated through published articles (*n* = 212, 75%), whereas the 110 studies reported from the USA were mostly communicated through proceedings (*n* = 60, 55%).

**Figure 2:**
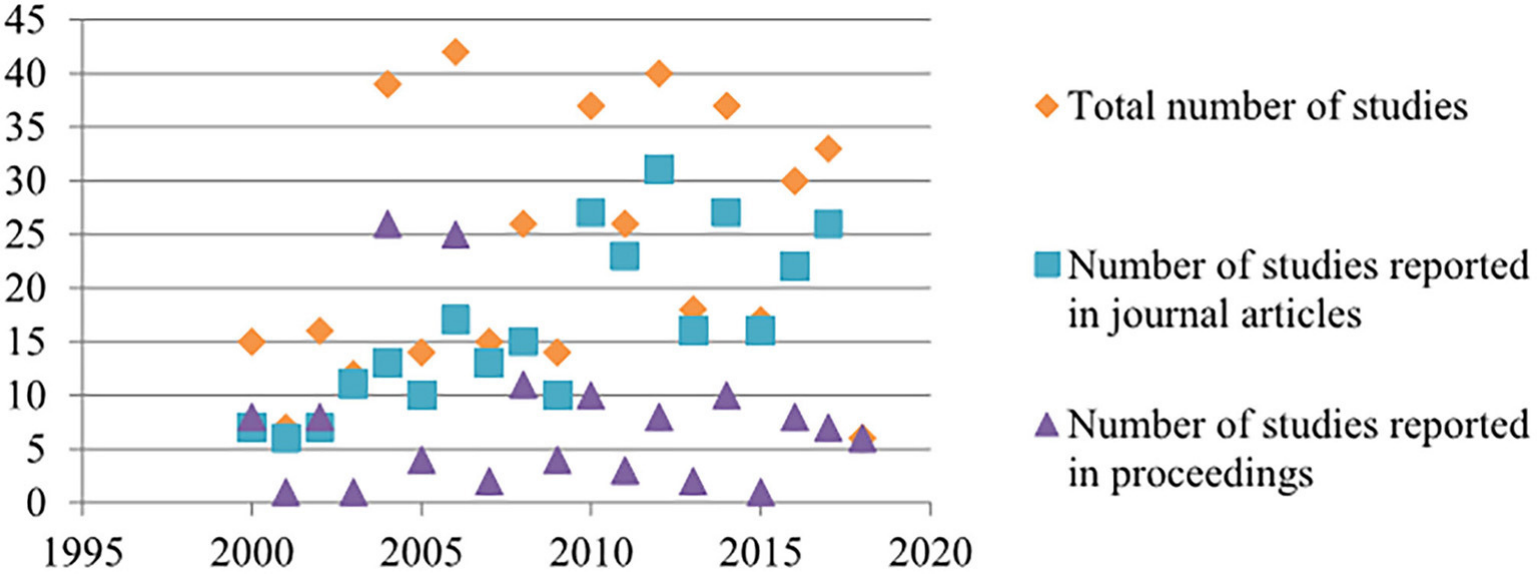
Annual number of included studies by study type from journal articles from 2000 to 2017 and proceedings from 2000 to 2018.

The annual number of included studies reported in published articles increased in an approximately linear trend since 2000, whereas the number of proceedings varied every other year in accordance with the alternate year schedule of the International Pig Veterinary Society Congress and the number of accepted proceedings at the congress and the Annual Meeting of the AASV (Figure 2).

### Synthesis of Results for Clinical Trials and Observational Studies

The stated purpose(s) of the intervention as disease prevention, disease control, and/or performance varied according to the farm settings among the 414 clinical trials and 27 observational studies. There were no studies that evaluated an intervention for the clearly stated purpose of treatment of individual sick pigs.

Studies where the purpose of the intervention was for disease control (*n* = 134) were conducted primarily on commercial farms (*n* = 122, 91%), whereas when the purpose of the intervention as prevention (*n* = 250), studies were conducted on both commercial farms (*n* = 75, 30%) and research farms (*n* = 153, 61%). Among the 414 clinical trials, 238 (57%) evaluated an intervention for the purpose of prevention of which the majority were an evaluation of vaccines in piglets and/or dams (*n* = 167, 70%). Although all included studies (*n* = 441) reported a health outcome of interest in nursery pigs, many clinical trials (*n* = 238, 57%) and some observational studies (*n* = 5, 19%) also reported performance as a purpose of the intervention.

#### Non-antibiotic Interventions or Risk Factors

All eligible studies measured a health outcome in nursery pigs; however, interventions were applied to one or more specific populations based on production stage comprising dams, suckling piglets, or nursery pigs. Some studies reported the application of the intervention to more than one population. Among the total populations described in the eligible studies (*n* = 553), interventions were most commonly applied to nursery pigs (*n* = 406, 73%), followed by suckling piglets (*n* = 86, 16%), and dams (*n* = 61, 11%). Some studies reported the application of the intervention to all three populations (*n* = 27).

**Figure 3:**
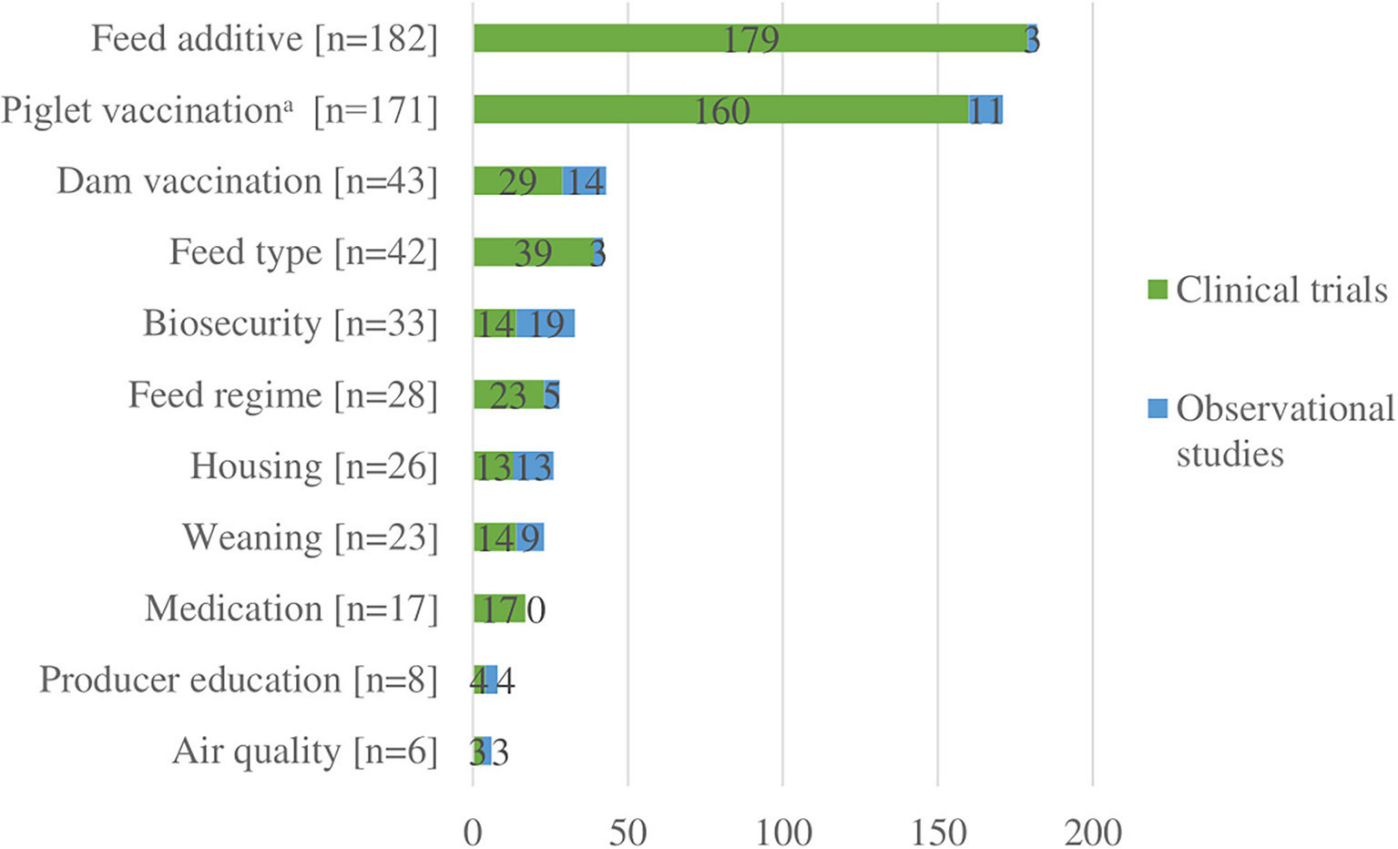
Number of interventions or risk factors (*n* = 579) described in clinical trials (*n* = 414) and observational studies (*n* = 27).

We charted data for 11 different categories of interventions or risk factors (Figure 3). Among the 414 clinical trial studies, there were 495 interventions described (Appendix 4). The two categories of interventions most frequently reported were feed additives (*n* = 179) and nursery or suckling piglet vaccination (*n* = 160) (Figure 3). Together, these two categories accounted for 68% of all interventions. The least common categories were air quality (*n* = 3) and producer education (*n* = 4). Details of all specific interventions for clinical trials are presented in Appendix 4; and further details for these specific interventions regarding comparison groups and health outcomes reported are available upon request. Among the 27 observational studies, there were 84 interventions or risk factors described (Table 4). The two categories that were most frequently studied were biosecurity (*n* = 19) and vaccinations of dams (*n* = 14).

**Table 4:** Risk factors described in observational studies (*n* = 27).

**Risk factors^**a**^ (*n* = 84)**	**Risk factor details**
Biosecurity (*n* = 19)	All-in-all-out vs. continuous flow (*n* = 7), mixing/cross fostering (*n* = 5), internal and external biosecurity (*n* = 5), air space separation (*n* = 2), piglet movement between stages (*n* = 3), infection control (*n* = 4), dead pig removal (*n* = 1)
Vaccination of dams (*n* = 14)	Porcine reproductive and respiratory syndrome virus (PRRSV) (*n* = 4), Porcine Circovirus type 2 (PCV2) (*n* = 3), Enterotoxigenic *Escherichia coli* (ETEC) (*n* = 3), rotavirus (*n* = 1), not clear or unspecified (*n* = 3)
Housing (*n* = 13)	Pen floor type (*n* = 4), space allowance/pig density (*n* = 4), use of bedding (*n* = 2), drinker type (*n* = 2), climatic and temperature conditions (*n* = 3), age of buildings (*n* = 1), indoors vs. outdoors (*n* = 1)
Vaccination of piglets^b^ (*n* = 11)	PRRSV (*n* = 4), PCV2 (*n* = 4), not clear, or unspecified (*n* = 5)
Weaning (*n* = 9)	Weaning age (*n* = 8), mixing at weaning or weaning management (*n* = 3)
Feed regime (*n* = 5)	Restricted feeding (*n* = 4), creep feeding (*n* = 3), starter diet protein content restriction (*n* = 1)
Producer education (*n* =4)	Experience level of manager/producer/worker (*n* = 3), Education level of manager/producer/worker (*n* = 2)
Feed type (*n* = 3)	Pelleted nursery feed (*n* = 1), feed composition quality (*n* = 1), level of soybean and canola (*n* = 1)
Feed additive (*n* = 3)	Zinc product (e.g., ZnO) (*n* = 3)
Air quality (*n* = 3)	Ventilation (*n* = 3)

*Categories of risk factors presented in order of decreasing frequency*.

a *Risk factors included modifiable exposures regardless of the positive or negative impact of the exposure on an outcome*.

b *Piglet vaccination includes suckling piglet or nursery pig vaccination*.

#### Interventions Evaluated as Comparison Groups

Among the clinical trials and the observational studies, there were 672 comparison groups described. The most frequently reported comparison group was “different form or level” of the intervention or exposure (*n* = 328, 49%), followed by a no treatment control group (*n* = 221, 33%). The clinical trials also described comparison groups that received placebo (*n* = 84, 13%), antibiotics (*n* = 24, 4%), ZnO (*n* = 8, 1%), or a combination product containing an antibiotic and ZnO (*n* = 3, <1%). Note that some studies compared a non-antibiotic intervention group with a ZnO comparison group, whereas other studies compared a ZnO treatment group with a no treatment control group, or antibiotic or other treatment comparison group.

Among the 27 clinical trials that included an antibiotic comparison group, 21 investigated various feed additives, three investigated vaccinations to control *Mycoplasma hyopneumoniae*, one investigated a feed type, one investigated housing at the time of weaning, and one investigated producer education in the form of individual pig care training vs. standard metaphylactic antibiotic use in cases of nursery pig morbidities (20) (Appendix 4). Among the 179 clinical trials that investigated feed additives, 10 included a ZnO comparison group (Appendix 4).

#### Outcomes Measured

**Figure 4:**
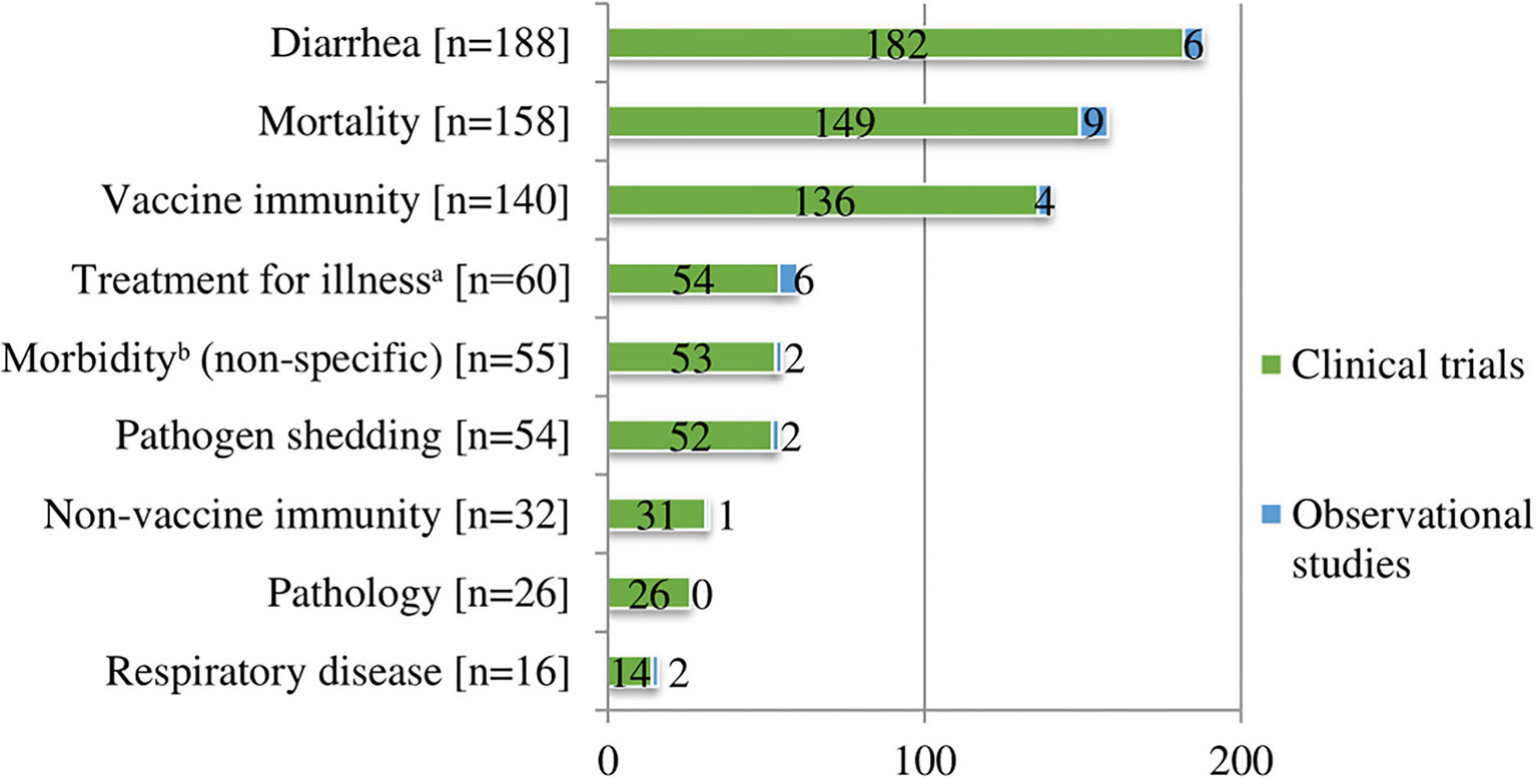
Number of health outcomes of interest^*a*^ (*n* = 729) described in clinical trials (*n* = 414) and observational studies (*n* = 27).

We charted data for nine health outcomes of interest. Among the clinical trials and observational studies, there were 729 reported outcomes (Figure 4). The three most commonly reported outcomes included clinical diarrhea (*n* = 188, 26%), mortality (*n* = 158, 22%), and vaccine immunity (*n* = 140, 19%). Immunity to vaccines included measures of specific immunity (*n* = 140) (e.g., serology and/or pathogen recovery or identification with PCR, and cell-mediated immunity) and in five studies also included non-specific immunity. Reporting of measures of immunity to a vaccine, with or without reporting other outcomes, was common among vaccine clinical trials (*n* = 118). A total of 43 (36%) vaccine clinical trials only reported measures of immunity without reporting any clinically important outcomes. These trials were conducted on research farms (*n* = 18), commercial farms (*n* = 20), and farm settings in which it was unclear (*n* = 5). Other outcomes reported included treatment for illness or antibiotic use (*n* = 60, 8%). Antibiotic treatments were typically for diarrhea when specified. The metric used for treatment for illness or antibiotic use varied (e.g., number of treatments, percent treated animals, treatment incidence calculated on an animal daily dose basis, and farm-level antibiotic use). Less commonly reported outcomes included non-diarrheal, non-respiratory, non-specific morbidity (e.g., fever, removals, or unthriftiness) or other morbidities such as lameness (*n* = 55, 8%) and pathogen shedding (*n* = 54, 7%). The remainder of the reported health outcomes of interest included non-vaccine immunity (*n* = 32, 4%), which comprised studies that measured specific immunity (*n* = 21), non-specific immunity (*n* = 10), or both (*n* = 1). Presence of pathological lesions (e.g., lung lesions at necropsy or injection site lesions) (*n* = 26, 3.5%) and clinical respiratory disease (*n* = 16, 2%) also were reported (Appendix 4).

Among the 441 included clinical trials and observational studies, all of which reported a health outcome of interest, the most commonly reported additional outcomes included performance outcomes such as growth (*n* = 297, 67%), feed efficiency (*n* = 197, 45%), and feed intake (*n* = 186, 42%).

#### Study Size

Among both clinical trials and observational studies, the study size varied widely from the smallest study using six individuals to evaluate an autogenous vaccine (21) to the largest study using 331,592 individual pigs to evaluate the impact of a producer education program on nursery pig mortality (22) (Table 5). Some studies measured health outcomes at the individual level but performance outcomes at the pen level. Most studies measured outcomes at the individual level regardless of the level of intervention allocation (Table 5). Among the observational studies alone, studies varied from 160 to 3,736 individuals.

**Table 5:** Study size^a^ of clinical trials and observational studies at the hierarchical level of the data analysis.

	**Number of studies**	**Range of study sizes**	**Experimental settings**	**Commercial settings**	**Unclear settings**
Individual	405	9–3,31,592	200	179	26
Group/pen/room	113	2–653	63	45	5
Herd	37	3–1,513	0	37	0

a *Study size was the number of study subjects included in the analyses*.

*Some studies measured outcomes at multiple levels*.

*For some studies some outcomes were measured at the individual level but performance was measured at the pen level*.

### Material for Potential Systematic Review Questions

There were 13 interventions evaluated in clinical trials that met our inclusion criteria for studies that could feasibly support systematic reviews; these included feed additives (e.g., amino acids, diet acidification, organic acids, fiber, phytobiotics, pre-biotics, probiotics, egg yolk antibodies, and ZnO), vaccination of piglets [e.g., porcine circovirus 2 (PCV2), porcine reproductive and respiratory syndrome virus (PRRSV), and *M. hyopneumoniae*], and vaccination of dams (e.g., PCV2) (Appendix 4).

### Knowledge Gaps

The selection of knowledge gaps identified discretionary items based on the authors' opinions. We found that there were relatively few studies in which a non-antibiotic intervention was compared directly with an antibiotic comparison group. Among the vaccine clinical trials, approximately one third failed to report a clinically important outcome.

## Discussion

This scoping review of non-antibiotic approaches for disease prevention or control that may reduce the need for antibiotics in nursery pigs relevant to the North American context identified a large body of literature with considerable breadth and depth. Since most of the studies described in this ScR were conducted in the EU or the UK, we may have not captured a body of knowledge on this broad topic for the North American context. The breadth of this literature was reflected in the diversity of interventions or risk factors evaluated, whereas the depth of this literature was reflected in the number of specific topic areas with similar studies that might feasibly support systematic reviews.

Clinical and policy decisions are generally regarded as best guided by the interpretation of findings of multiple studies evaluating the same research question rather than the findings of a single study, which is a random event from a distribution of possible results (23). A summary of multiple relevant studies in the form of a systematic review can provide a credible summary of the primary literature (23). However, a third of the literature included in this scoping review was sourced from proceedings that present at least three challenges to systematic reviews. First, proceedings obtained through the AASV Swine Information Library were available exclusively to members and thus would not be available to review teams through database searches. Second, these proceedings databases were not searchable through word string searches, which are an efficient method to search the literature on a specific topic. Third, these proceedings were not peer-reviewed and often were short; thus, the quality of research reporting in proceedings may be insufficient for inclusion into systematic reviews. Brace et al. (24), in an evaluation of the quality of reporting of vaccine trials at veterinary conferences, concluded that it would be difficult to assess validity from the information provided in most conference proceedings. Although there was apparently considerable depth in this body of literature, without the inclusion of proceedings, the depth may actually be considerably less. Interestingly, the majority of research from the USA on this broad topic was available only through proceedings. Similarly, Brace et al. (24) reported that only 6% of 89 proceedings presented on swine vaccines at the AASV annual conference from 1988 to 2003 were later published as full articles. Although assessing the proceedings-to-publication ratio from AASV conferences was not our objective, our findings of only two duplicate journal articles with conference proceedings suggest the ratio is still low. This may represent a lost opportunity for the communication of research.

We chose to focus our data charting on controlled clinical trials. We did not include quasi-experimental trial design (i.e., before and after intervention comparisons), as this design does not provide an equal evidentiary value to clinical trials (19). Though challenge trials serve an important purpose in providing proof of concept prior to field trials under natural exposures, challenge trials tend to report more favorable outcomes compared with clinical trials of the same intervention (25). We found that the body of literature in this review was dominated by clinical trials with comparatively few observational studies. This may reflect the comparative ease of conducting and study design appropriateness of clinical trials vs. observational studies on swine farms.

This scoping review identified a wide variety of vaccines and feed additives, which together comprised the majority of interventions evaluated in clinical trials. There was a dearth of clinical trials that evaluated management interventions such as biosecurity and infection control, feed or nutrient restrictions, housing, and weaning. This may reflect the difficulty of evaluating these types of interventions in clinical trial settings and/or the difficulty in sourcing funding for trials of these interventions (26). Biosecurity was the most frequently studied intervention among the relatively low number of observational studies identified by this scoping review. Thus, the depth of research available for synthesis on management practices was far less than for other interventions.

Despite the approximately equal number of studies conducted on research farms vs. commercial farms, there was a strong predominance of research with the purpose of preventing disease on research farms, whereas research with the purpose of disease control was predominately conducted on commercial farms. Variables that may impact the outcome of a trial, such as prior disease-free status, could potentially be better controlled on research farms than commercial farms, whereas the field conditions of commercial farms, such as an existing disease problem, provides a better setting to truly test the effectiveness of an intervention under “natural commercial” conditions (27). However, it is unknown to what extent the research vs. commercial farm settings impact external validity in swine research.

Among the studies identified through this scoping review, approximately half reported a comparison group that was a different form or level of the intervention itself. Traditional systematic reviews and meta-analyses are not just based on studies with similar interventions, populations, and outcomes but also on similar comparison groups. In the absence of sufficient studies with similar comparison groups, combining studies with the same outcome through network meta-analysis may prove useful. Network meta-analysis allows comparisons of interventions that may not have been directly compared in head-to-head trials by mathematically evaluating both direct and indirect comparison evidence of multiple interventions and comparisons (28). If we had restricted our scoping review to studies that compared a non-antibiotic intervention group with an antibiotic intervention group, our review would have been very limited: first, because most of these studies evaluated a feed additive intervention, and second, because there were relatively few feed additive intervention studies with an antibiotic comparison group. In a body of literature that describes non-antibiotic approaches to improve the health of nursery pigs, this lack of comparisons with antibiotics may represent a knowledge gap for decisions about antibiotic alternatives. Where it may be appropriate to use an antibiotic comparison group, the results could potentially demonstrate the superiority or at least the non-inferiority of a non-antibiotic intervention.

Approximately one third of vaccine clinical trials did not report a clinically important outcome, though they did report measures of immunity. The lack of reporting clinically important outcomes when they could have been measured reduces our opportunity to build a body of evidence best suited for clinical decision making. Clinically important outcomes as determined by guidelines, clinicians, patients, or the researcher provide the best evidence for inclusion in systematic reviews, whereas indirect outcomes such as measures of immunity provide a lower quality of evidence (29–33). To enhance research efficiency, future vaccine clinical trials should report clinically important outcomes.

Beyond describing the body of literature pertaining to non-antibiotic approaches that may reduce the need for antibiotics for disease prevention or control in nursery pigs, an additional objective of this review was to identify specific topic areas where there may be sufficient literature to support systematic reviews. We identified 13 specific topic areas with a minimum of 10 clinical trials that may feasibly support systematic reviews. These topic areas were composed of various vaccines and feed additives. Although we listed the ZnO as an intervention for which there may be sufficient material to support an SR, we do not recommend knowledge synthesis for this intervention given that concerns regarding AMR co-selection with the use of ZnO in swine (34). Though this scoping review identified numerous specific topic areas that might be feasibly combined in systematic reviews, similarity among comparison groups and choice of outcome would need to be carefully considered. Nevertheless, systematic reviews of these topic areas for nursery pigs, if not already conducted, could provide a useful synthesis of existing knowledge. Some systematic reviews of related topics have been conducted (35–39); however, none of these systematic reviews pertained exclusively to health outcomes in nursery pigs.

In determining the specific topic areas with sufficient similar studies to support systematic reviews, we used an arbitrary number of 10 similar clinical trials with some commonality of the intervention and population. Technically, a minimum of two studies are all that is needed for combination in a meta-analysis if those studies are similar enough to combine in a meaningful way (40). However, having additional studies provides an opportunity to explore between-study variability, which in turn impacts the interpretation and meaning of the meta-analysis (40), (41).

There were potential limitations that may have impacted the comprehensiveness of this scoping review. First, we may have missed some articles if we did not include all possible terms for each of the many non-antibiotic interventions included in the search. Systematic reviews for specific interventions should maximize comprehensiveness by including all possible terms. Second, we may have overlooked some interventions or outcomes if they did not appear in the title or abstract. Third, we accessed bibliographic sources available through the University of Guelph data platforms and two conference proceedings available through the AASV library. We may have missed additional published articles available through other databases, and unpublished studies generated by companies testing products or proceedings from other conferences. Fourth, our search using the CAB Direct platform was unsuccessful due to technical difficulties. Without the additional studies identified through that platform, our search may not have been as comprehensive as we had intended. The coverage provided by CAB Abstracts was found to be excellent in a comparison of nine databases for veterinary journals (42), so it is possible that the CAB platform contained relevant articles that our search did not identify. Finally, due to limited resources, we could not include all possibly relevant sources of gray literature such as symposia proceedings. We chose to focus on the gray literature of North America.

In addition to limitations to comprehensiveness, this review may have two additional limitations. First, we may have misclassified the purpose of the intervention for disease control by including in this category pig herds or groups that also had a known exposure to an infection and not solely groups that contained clinically ill or infected pigs as defined by AVMA and GAO reports. Finally, we did not assess inherent biases of included studies such as lack of appropriate randomization of clinical trials, lack of concealment or blinding, loss to follow-up, or selective outcome reporting. Any systematic reviews of non-antibiotic approaches that may reduce the need for antibiotic prevention or control in nursery pig production should include a risk of bias assessment (43).

## Data Availability Statement

The raw data supporting the conclusions of this article will be made available by the authors, without undue reservation.

## Author Contributions

LW coordinated the project, acted as the primary reviewer for relevance screening and data charting, analyzed data, interpreted the results, and wrote the manuscript drafts. JS oversaw the work, piloted the relevance screening and data charting forms, assisted with the interpretation of results, reviewed manuscript drafts, and approved the final manuscript. AO'C and SM provided guidance for interpretation of the results, commented on the manuscript drafts, and approved the final manuscript. TO'S provided guidance for the stakeholder survey and interpretation of the results, commented on the manuscript drafts, and approved the final manuscript. MR and KC conducted relevance screening and data charting as a second reviewer, commented on manuscript drafts, and approved the final manuscript version. All authors contributed to the article and approved the submitted version.

## Conflict of Interest

The authors declare that the research was conducted in the absence of any commercial or financial relationships that could be construed as a potential conflict of interest.
