# Supplementary material for: Non-antibiotic Approaches for Disease Prevention and Control in Nursery Pigs: A Scoping Review
Source: Front Vet Sci. 2021 Apr 21;8:620347. doi: 10.3389/fvets.2021.620347 (PMC8097137; doi:10.3389/fvets.2021.620347)
Supplement: Supplementary file 1 [file Data_Sheet_1.docx]

**Appendix 1.** Relevance Screening forms from Level 1–4 for Scoping Review of non-antibiotic approaches in nursery pigs.

**Level 1 Relevance Screening**.

| 1. | **Hypothesis testing study** involving live pigs: clinical trial, challenge trial, or analytical observational study? [choose one] |
| --- | --- |

Yes

Unclear

No [and SUBMIT]

| 2. | Does this study report an outcome measured in **nursery stage pigs**? [choose one] |
| --- | --- |

Yes

Unclear

No [and SUBMIT]

| 3. | Does this study report an **intervention or management practice** of interest? [choose one] |
| --- | --- |

Yes

Unclear

No [and SUBMIT]

| 4. | Does the study measure a **health or performance outcome** of interest? [choose one] |
| --- | --- |

Yes

Unclear

No [and SUMBIT]

| 5. | Was this study conducted in a **country/region** of interest? [choose one] |
| --- | --- |

Yes or Unclear

ignore this button

No [and SUBMIT]

**Level 2 Relevance screening by country of interest**

| 1. | Does the first author address correspond to a country of interest?  [Canada, USA, Western EU [include Poland, Hungary, and Czech Republic] Britain, Australia/NZ] |
| --- | --- |

Yes

Unclear

No

**Level 3** **Relevance screening by study design and type of pig population**

| 1. | What type of study is this? | |
| --- | --- | --- |
| Challenge trial [list agent or pathogen] [i.e., enterotoxigenic E. coli, ovalbumin, etc.] EXCLUDED | |  |
| Clinical trial_ INCLUDED | |  |
| Both challenge AND clinical trial [list challenge agent] INCLUDED | |  |
| Observational study INCLUDED | |  |
| Unclear INCLUDED | |  |
| 2. | What type of pigs? | |

| Conventional INCLUDED |  |
| --- | --- |
| SPF [specific pathogen free] INCLUDED |  |
| Gnotobiotic / cesarean born / germ free EXCLUDED |  |
| Cannualted / metabolic chamber EXCLUDED |  |
| Transgenic EXCLUDED |  |
| Conventional or SPF + other [please list] INCLUDED |  |

  **Level 4 Relevance screening by intervention and outcome of interest**

Top of Form

Bottom of Form

| 1. | Does the study report on interventions of NO interest? [EXCLUDE]  1. Interventions to mitigate feed mycotoxins (Fusarium toxins, Aflatoxin, CON, ZON, etc.]  2. Interventions to mitigate soy allergens | |
| --- | --- | --- |
| No, some other NON-ANTIBIOTIC intervention [INCLUDE] | |  |
| Yes intervention to mitigate feed mycotoxins [EXCLUDE] | |  |
| Yes intervention to mitigate effects of soy allergen [EXCLUDE] | |  |
| 2. | Does this study evaluate an intervention to prevent or treat a disease of NO interest? | |

| No, some other disease or performance outcome [INCLUDE] | |  |
| --- | --- | --- |
| Yes, Classic Swine Fever (CSF) [EXCLUDE] | |  |
| Yes, pseudorabies aka Aujesky's disease [EXCLUDE] | |  |
| Yes, Foot and mouth disease (FMD) [EXCLUDE] | |  |
| Yes, Hepatitis E [EXCLUDE] | |  |
| Yes, methicillin resistant Staph. aureus (MRSA) [EXCLUDE] | |  |
| 3. | Does this citation report ONLY a performance outcome? | |

| No, THERE IS a CLINICALLY IMPORTANT health outcome reported [diarrhea, scours,f ecal consistency or fecal score, morbidity, mortality, clinically important infections, shedding clinically important pathogens [Salmonella, Campylobacter, Enterotoxogenic E.coli or any E.coli linked to diarrhea, immunity for vaccines/bacterins] [INCLUDE] |
| --- |
| Yes, ONLY performance outcomes reported or other health outcome EXCLUDE |

**Appendix 2.** Level 5: Data Charting form for Scoping Review of non-antibiotic approaches in nursery pigs.

| 1. | Does this reference/citation meet level 1-4 inclusion criteria?  i.e., intervention of interest, in country of interest, health outcome of interest measured in nursery stage piglets. | |
| --- | --- | --- |
| Yes | |  |
| No, not relevant [SUBMIT] [see previous exclusions] | |  |
| No, not the right country/region [SUBMIT] | |  |
| No, outcome is performance or 'other health' outcome ONLY [SUBMIT] | |  |
| 2. | What is the reference/citation type? | |

| Full text article | |  |
| --- | --- | --- |
| Full text proceeding [e.g., short research paper cited in association with a conference or meeting), research report, or product report | |  |
| Full text thesis | |  |
| Abstract only [SUBMIT] | |  |
| [Clear Response](https://v2dis-prod.evidencepartners.com/Submit/ScreenArticles.php?formid=5&levelid=5&refid=74507&set_id=21530) | | |
| 3. | How many RELEVANT studies are included in this reference?  [relevant based on Level 1-4 criteria]  Add a comment if needed for clarification. | |

| 1 | |  |  |
| --- | --- | --- | --- |
| 2 | |  |  |
| 3 | |  |  |
| 4 | |  |  |
| 5 | |  |  |
| 6 | |  |  |
|  | | |  |
| 4. | To which RELEVANT study does this form apply?  Use the order as they appear in the M&M. | | |

| # 1 | |  |  |
| --- | --- | --- | --- |
| # 2 | |  |  |
| # 3 | |  |  |
| # 4 | |  |  |
| # 5 | |  |  |
| # 6 | |  |  |
|  | | |  |
| 5. | In what country/region did this study take place? | | |

Canada [list province code]

USA [list state code]

EU [list country]

Australia/New Zealand

Unknown

| 6. | What type of study design was used?  [choose all that apply] | |
| --- | --- | --- |
| Clinical trial | |  |
| Observational study | |  |
| 7. | Year of publication? | |

| 8. | What was the sample size at the level of which the outcome was analyzed?  [choose all that apply] | | |
| --- | --- | --- | --- |
| # Individuals | |  |  |
| # Pens/groups/litters | |  |  |
| # Herds or farms | |  |  |
| 9. | Was the population an experimental or commercial population?  [choose all that apply] | | |

| Experimental [living on a university or pharmaceutical company farm] | |  |
| --- | --- | --- |
| Commercial [explicitly stated as commercial conditions] | |  |
| Unclear | |  |
| 10. | What was the purpose of the intervention?  [choose all that apply] | |

| Prevent disease [e.g., improve immunity or gut function, reduce stress or exposure, etc.] | |  |
| --- | --- | --- |
| Treat disease [e.g., given after exposure to mitigate clinical disease] [List disease] | |  |
| Improve performance | |  |
| 11. | At what stage(s) was the intervention administered?  [choose all that apply] | |

| Nursery | |  |
| --- | --- | --- |
| Suckling | |  |
| Sow/gilt | |  |
| 12. | What intervention/management practice or risk factors were studied?  [choose all that apply] | |

| Vaccination to piglet [please list] | |  |
| --- | --- | --- |
| Maternal vaccination [please list] | |  |
| Feed or water additive (non-antibiotic) [e.g., amino acids, probiotics, feed enzymes, plant extracts, zinc or copper, etc.] [please list] | |  |
| Medication (non-antibiotic medication administered directly to the pig by injection or orally, etc.] [please list] | |  |
| Feeding regime [e.g., creep feed, feed restriction, protein restriction, etc.] [please list] | |  |
| Diet type or format [e.g., distillers grains, pelleted or liquid feed, complexity, etc.] [please list] | |  |
| Weaning method [age at weaning, mixing litters, intermittent suckling, etc.] [please list] | |  |
| Biosecurity or bio-containment [exposures from other farm, comingling from off farm, comingling within farm, introductions, disease eradication, pig flow, all-in-all-out, etc.] [please list] | |  |
| Housing, flooring, feeders [please list] | |  |
| Air quality [please list] | |  |
| Producer education [please list] | |  |
| Other or clarification [please list] | |  |
|  | | |
| 13. | What comparison group(s) was used?  [choose all that apply] | |

| no treatment | |  | |
| --- | --- | --- | --- |
| placebo or sham treatment | |  |  |
| different treatment or different level of treatment or exposure level [please list] | |  |  |
| antibiotic [please list] | |  |  |
| zinc oxide [please list] | |  |  |
| 14. | What health outcome was measured?  [choose all that apply] | | |

| Mortality | |  |
| --- | --- | --- |
| Diarrhea/fecal score or consistency/scours/dysentery | |  |
| Respiratory disease | |  |
| Fecal shedding of a pathogen [please list pathogen] | |  |
| Immunity to a vaccine or bacterin [e.g., virology, serology, cell mediated immunity, viremia, other] [please list] | |  |
| Immunity related to a non-vaccine intervention | |  |
| Morbidity [unspecified or a non-diarrhea or non-respiratory condition [please list] | |  |
| Antibiotic usage or # treatments | |  |
| Pathology [lesions] | |  |
| Other [please list] | |  |
| None [no health outcome measured] | |  |
| 15. | What production, or other type of health, outcome was measured?  [choose all that apply] | |

| None [no performance or 'other health' OC measured] | |  | |
| --- | --- | --- | --- |
| Performance [feed intake, ADFI] | |  | |
| Performance [growth, ADG, body wt.] | |  | |
| Performance [efficiency, G:F ratio] | |  | |
| Farm economics, treatment costs [please list] | |  |  |
| Diet digestibility | |  |  |
| GI microflora | |  |  |
| GI morphology | |  |  |
| 16. | Comments if needed. | | |

**Appendix 3.** Description of pathogen and immune agents used in challenge trials (n=536) for Scoping Review of non-antibiotic approaches in nursery pigs.

| Challenge agents | Number of challenge trials^a^ |
| --- | --- |
| Pathogens | |
| Enterotoxigenic *Escherichia coli* (ETEC):  K88 (n=43), O149 (n=13), unspecified (n=72) | 128 |
| Porcine reproductive and respiratory syndrome virus (PRRSV) | 78 |
| Porcine circovirus type 2 (PCV2) | 58 |
| swine influenza A virus (IAV) previously known as SIV | 52 |
| *Salmonella* spp.:  *Salmonella typhimurium* (n=35), *Salmonella choleraesuis* (n=7), unspecified (n=2) | 44 |
| *Mycoplasma hyopneumoniae* | 39 |
| *Lawsonia intracellularis* | 23 |
| *Haemophilus parasuis* (now known as *Glaesserella parasuis* (GPS)) | 14 |
| *Streptococcus suis* (*S. suis*) | 10 |
| *Actinobacillus pleuropneumoniae* (APP) | 6 |
| Porcine epidemic diarrhea virus (PEDV) (coronavirus) | 5 |
| *Bordetella bronchiseptica* | 3 |
| *Brachyspira hyodysenteriae* | 2 |
| Immune agents | |
| Lipopolysaccharide (LPS), *E. coli* endotoxin | 23 |
| Ovalbumin | 6 |
| Keyhole limpet haemocyanin (KLH) | 4 |
| Tetanus toxoid | 3 |

^a^ Some studies described more than one challenge agent

**Appendix 4**. Interventions described in clinical trial studies (n=414) for Scoping Review of non-antibiotic approaches in nursery pigs. Categories of interventions presented in order of decreasing frequency. Some studies included multiple interventions.

| Intervention by categories (n=495) | Intervention details (some studies described more than one type of intervention within a category) | Farm setting |
| --- | --- | --- |
| Feed additive (n=179) | Amino acids (n=14), acidification (n=7). Organic acids (n=10), algae/seaweed /seaweed extracts [laminarin and fucoidan] (n=7), clinoptilolite (n=4), colostrum (n=2), copper (n=5), enzymes (n=2), fermentable protein (n=1), fiber (n=10), flavor (n=1), nucleotide (n=1), medium chain triglycerides (MCT) (n=1), phytobiotics (n=10), prebiotics (n=17), probiotic (n=8), synbiotics (n=2), egg yolk antibodies (n=2), spray-dried plasma (n=3), sweetener (n=1), yeast (n=5), zinc product (Zn^a^) (n=15) | Experimental  (n=124) |
|  | Acidification (n=4), amino acids (n=3), egg yolk antibodies (n=8), electrolytes (n=1), enzymes (n=2), fatty acids (n=1), fish oil (n=1), iodine (n=1), organic acids (n=3), phytobiotics (n=6), prebiotics (n=1), probiotics (n=8), spray dried plasma (n=3), Vit. E (n=2), Zn product (n=4), Cu (n=1) | Commercial (n=48) |
|  | Prebiotic (n=1), probiotic (n=1), acidification, organic acids (n=1), nucleotide (n=1), fatty acids [MCFA] (n=2), Copper (n=1) | Unclear  (n=7) |
| Vaccination of piglets (n=160) | Reproductive and Respiratory Syndrome Virus (PRRSV) (n=9), Porcine rhusiopathiae type 2 (PCV2) (n=3), *Actinobacillus pleuropneumoniae* (APP) (n=2), adenovirus serotype 5 (n=1), *Clostridium difficile* (n=2), Enterotoxigenic *Escherichia coli* (ETEC) (n=7), *Erysipelothrix rhusiopathiae_)_* (n=1), *Glaesserella parasuis* (GPS) (previously known as *Haemophilus parasuis* (Hps)) (n=2), *Lawsonia intracellularis* (n=3), *Mycoplasma hyopneumoniae* (*M. hyo*) (n=2), Porcine epidemic diarrhea virus (PEDC) [coronavirus] (n=1), *Salmonella typhimurium* (n=1), swine influenza A virus (IAV) (previously known as swine influenza virus (SIV) (n=8), *Streptococcus suis* (n=1) | Experimental (n=42) |
|  | PCV2 (n=37), *M. hyo* (n=33), PRRSV (n=14), ETEC (n=6), *Lawsonia intracellularis* (n=5), *Histophilus somni* (n=1), APP (n=1), *Salmonella typhimurium* (n=2), *Salmonella choleraesuis* (n=1), *Erysipelothrix rhusiopathiae (E. rhusiopathiae)* (n=1), GPS (n=9), *Clostridium* spp. (n=1), unspecified (n=4) | Commercial (n=104) |
|  | PCV2 (n=6), IAV (n=4), APP (n=2), *Clostridium perfringens* (n=1), *Lawsonia intracellularis* (n=1) | Unclear  (n=14) |
| Feed type (n=39) | Fermented soybean meal (n=5), pelleted vs. mash (n=2), oat hulls vs. main cereal (n=2), high quality vs. low quality (n=2), non-fermented soybean products, soybean meal or concentrate (n=8), diet complexity (n=1), dry feed vs. wet feed (n=1), Australian sweet lupins (n=1), cooked white rice (n=1), barley, wheat, corn, mixture (n=1), diet type with or without enzyme supplementation (n=1), heat processed maize vs. animal origin ingredients (n=1), starfish meal (SM) vs. fish meal (FM) vs. extruded soybean meal (ESBM) (n=1) | Experimental (n=27) |
|  | Diet complexity (n=4), rice vs. corn or other grains (n=3), pelleted vs. meal (n=1), dry vs. gruel creep feed (n=1), heat treated wheat (n=1), unspecified (n=1) | Commercial (n=11) |
|  | home mixed vs. pelleted diet (n=1) | Unclear  (n=1) |
| Vaccination of dams (n=29) | IAV (n=2), GPS (n=1), *E. rhusiopathiae* (n=1), ETEC (n=1) | Experimental (n=5) |
|  | PCV2 (n=10), M. hyo (n=4), GPS (n=4), *S. suis* (n=1), IAV (n=1), *S. typhimurium* (n=1), unspecified (n=1) | Commercial (n=21) |
|  | *Bordetella bronchiseptica* - *Pasteurella multocida* (n=1), IAV (n=1), PVC2 (n=1) | Unclear  (n=3) |
| Feed regime (n=23) | Protein level (n=8), feed restriction (n=3), creep feeding (n=2), fiber level (n=1), duration of feeding high quality vs. low quality (n=1) | Experimental (n=15) |
|  | Protein level (n=1), feed restriction (n=2), fiber level (n=1), creep feed (n=1) | Commercial (n=5) |
|  | Creep feed (n=1), Zn deficiency (n=1), supplemental milk to suckling piglets (n=1) | Unclear  (n=3) |
| Medication (n=17) | Hormone -glucagon-like peptide 2 (GLP-2) (n=4), garlic-derived diallyl disulfide (DADS) and diallyl trisulfide (DATS) (n=2), Beta-Glucan extract (n=1), chlorate-nitrate-lactate (chlorate) orally (n=1), *Bacillus pumilus* WIT 572 from seaweed (n=1), Loperimide (n=1), phytohemagglutinin (PHA) (n=1) | Experimental (n=11) |
|  | Antiserum (n=3), acetaminophen (n=1), hormone -glucagon-like peptide 2 (GLP-2) (n=1), polyoxyethylene and polyoxypropylene nonionic block copolymers (POE-POP) (n=1) | Commercial (n=6) |
|  |  | Unclear  (n=0) |
| Biosecurity (n=14) | Mixing litters (n=1), infection control [cleaning] (n=9) | Experimental (n=10) |
|  | Mixing litters (n=3), internal and external biosecurity (n=1) | Commercial (n=4) |
|  |  | Unclear  (n=0) |
| Weaning (n=14) | Weaning age (n=4), mixing at weaning (n=1), moving at weaning (n=1), abrupt vs. gradual (n=1), pen enrichment pre and post weaning (n=1) | Experimental (n=8) |
|  | Weaning age (n=5), gilt and sow litters weaned and kept separately (n=1) | Commercial (n=6) |
|  |  | Unclear  (n=0) |
| Housing (n=13) | Multi-litter housing system vs. conventional single-litter housing pre and post weaning (n=1), separate zones or open mixed zones (n=1), pen enrichment (n=2), indoors vs. outdoors (n=1), flooring (n=1), feeder space allowance (n=1) | Experimental (n=7) |
|  | Pig density (n=3), pen size at same pig density (n=1), nursery pens with open or solid partitions (n=1), unspecified (n=1) | Commercial (n=6) |
|  |  | Unclear  (n=0) |
| Producer education (n=4) |  | Experimental (n=0) |
|  | Individual pig care approach (IPC) (n=4) | Commercial (n=4) |
|  |  | Unclear  (n=0) |
| Air quality (n=3) | Airborne dust and ammonia (n=1) | Experimental (n=1) |
|  | Climactic conditions (n=1), electrostatic particle ionization system (n=1) | Commercial (n=2) |
|  |  | Unclear  (n=0) |

^a^ Zn products were charted as an intervention when stated as an intervention of interest of the study and as a comparison treatment when compared to a different intervention of interest.
